# Equal stroke volumes, different costs: left ventricular 4D flow in normal and failing hearts

**DOI:** 10.1186/1532-429X-14-S1-W22

**Published:** 2012-02-01

**Authors:** Jonatan Eriksson, Ann Bolger, Tino Ebbers, Carl Johan Carlhall

**Affiliations:** 1 Department of Medical and Health Sciences, Division of Cardiovascular Medicine, Linköping University, Linköping, Sweden; 2 Center for Medical Image Science and Visualization (CMIV), Linköping University, Linköping, Sweden; 3 Department of Medicine, Division of Cardiology, University of California San Francisco, San Francisco, CA, USA; 4 Department of Clinical Physiology, Linköping University Hospital, Linköping, Sweden

## Summary

Although total left ventricular (LV) stroke volume (SV) is equal in healthy subjects and heart failure patients with mild LV remodeling, the SV’s transventricular flow paths to ejection and diastolic energetics are different. These flow-specific markers of inefficiency of the failing heart are detectable prior to clinical decompensation, and might be predictors of progressive adverse cardiac remodeling.

## Background

Heart failure is a common disorder with a dismal prognosis. Cardiac remodeling is a key component of heart failure that progresses from adaptive to maladaptive as the disease worsens, and is associated with increased risks of symptoms and mortality. In earlier stages of heart failure with adaptive remodeling, patients may remain clinically compensated and the failing heart’s left ventricular (LV) stroke volume (SV) remains the same as in the normal heart. Recently, time-varying and complex three-dimensional flow patterns and energetics within the normal LV have been demonstrated. Based on such 4D flow-specific measures, we hypothesized that while the SV of normal and failing LVs may be equal, the SV’s diastolic kinetic energy (KE) and transventricular routes to ejection are different in the two states.

## Methods

**Figure 1:**
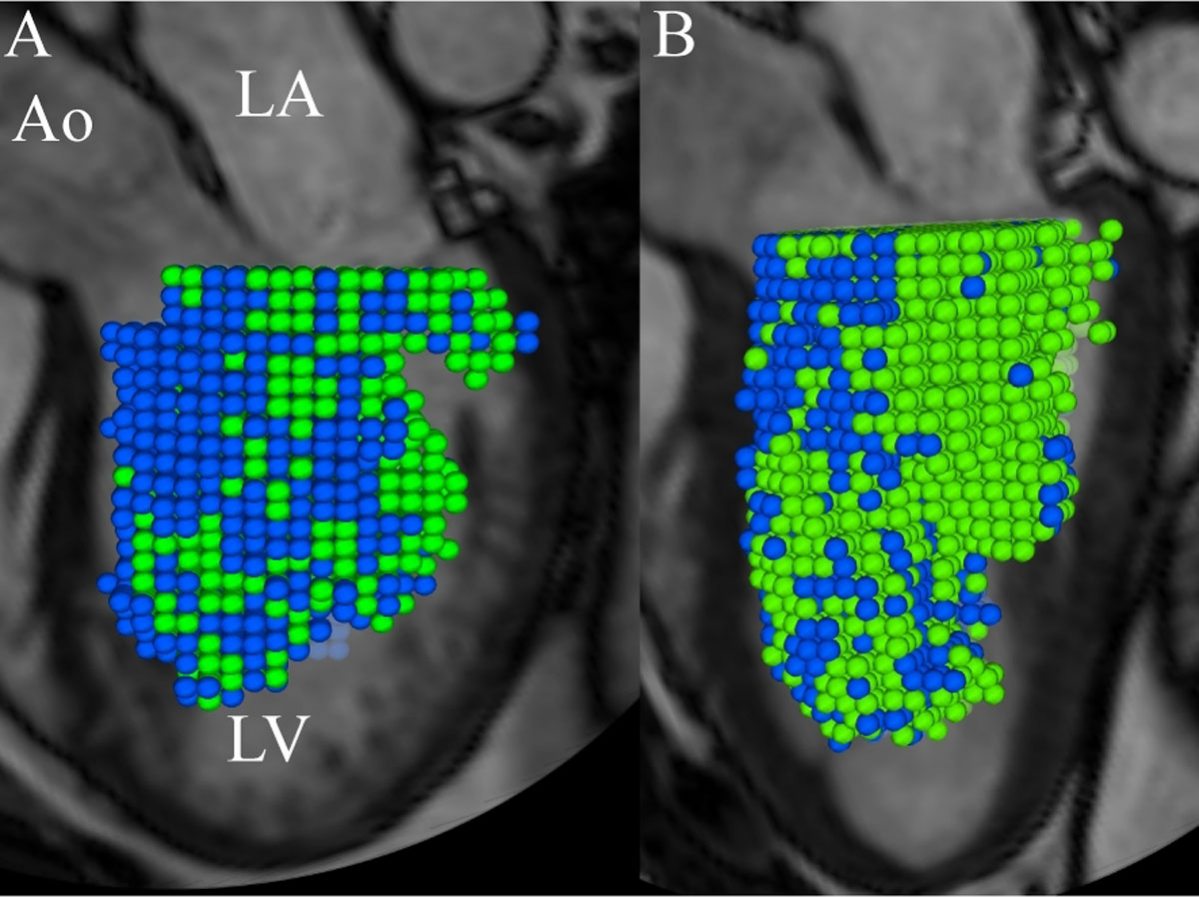
Pathline visualization at end-diastole of left ventricular Total stroke volume (SV) separated into Direct SV (green color) and Delayed SV (blue color), respectively. A) 62 y.o. patient with dilated cardiomyopathy; B) 42 y.o.healthy subject. Semi-transparent three-chamber image provides anatomical orientation. Ao, aorta; LA, left atrium; LV, left ventricle.

In ten patients with dilated cardiomyopathy (DCM) (6 females, 49±14 years [mean±SD]) and ten healthy subjects (4 female, 44±17 years), 4D phase-contrast CMR velocity data and morphological data were acquired at 1.5T (Philips Achieva). A previously validated method was used for the analysis: The LV endocardium was segmented from the morphological images at end-diastole (ED) and end-systole. Pathlines were emitted from the ED blood volume and traced forward and backward in time until end-systole, allowing separation of the Total SV into two flow components (figure 1): 1) Blood that enters and leaves the LV during the analyzed cardiac cycle (Direct SV), and 2) Blood that leaves but does not enter the LV during the analyzed cardiac cycle (Delayed SV). The KE was calculated over the cardiac cycle for these flow components based on the volume occupied by each pathline, its velocity, and blood density.

## Results

**Figure 2:**
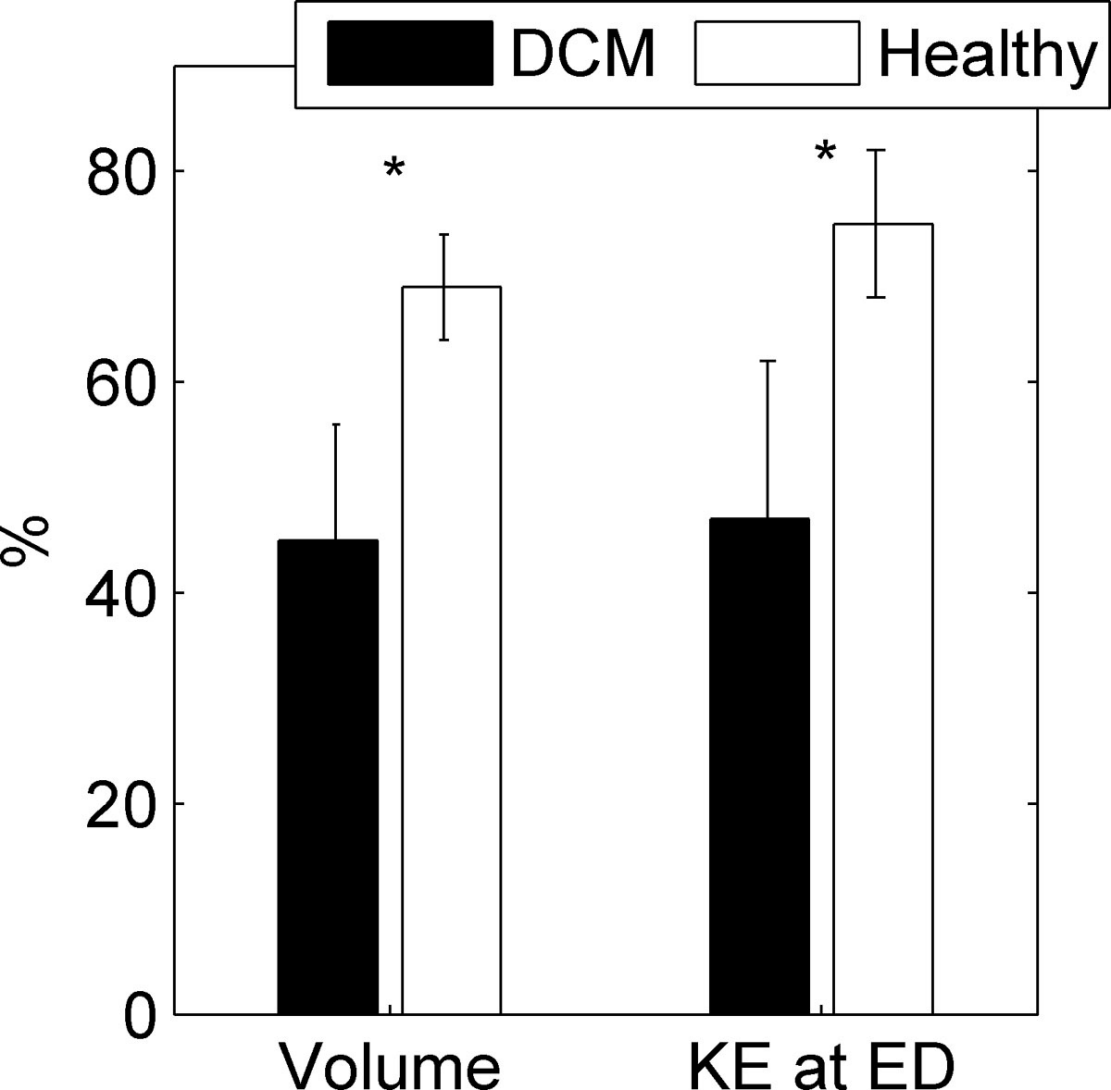
Left ventricular Direct stroke volume / Total stroke volume -ratios for volume (left) and kinetic energy (KE) at end-diastole (ED) (right). DCM, dilated cardiomyopathy. *p<0.001 vs healthy subjects.

The LV ED volume was larger and LV ejection fraction was smaller in DCM compared to healthy (179±33 vs 147±22 ml, p=0.021, and 42±5 vs 54±6 %, p<0.001, respectively). While the Total SV was equal in the two groups (77±19 vs 79±16 ml, p=0.8), the Direct SV represented a significantly smaller part of the Total SV in DCM compared to healthy (45±11 vs 69±5 %, p<0.001) (figure 2). At ED, the “KE of the Direct SV” / “KE of the Total SV”-ratio was lower in DCM compared to healthy (47±15 vs 76±7 %, p<0.001) (figure 2).

## Conclusions

Although total LV stroke volume is equal in healthy subjects and in clinically compensated heart failure patients with mild LV remodeling, the diastolic routes through the LV and pre-systolic energetics of the SV are significantly different in the two states. Inefficient flow may augment the chronic stress on the failing heart’s reserve, promoting progressive maladaptive cardiac remodeling.

## Funding

The Swedish Research Council

